# Variable repeats in the eukaryotic polyubiquitin gene *ubi4* modulate proteostasis and stress survival

**DOI:** 10.1038/s41467-017-00533-4

**Published:** 2017-08-30

**Authors:** Rita Gemayel, Yudi Yang, Maria C. Dzialo, Jacek Kominek, Jakob Vowinckel, Veerle Saels, Leen Van Huffel, Elisa van der Zande, Markus Ralser, Jan Steensels, Karin Voordeckers, Kevin J. Verstrepen

**Affiliations:** 1 Laboratory of Systems Biology, VIB Center for Microbiology, Leuven, B-3001 Belgium; 2 Laboratory for Genetics and Genomics, Center of Microbial and Plant Genetics (CMPG), Department M2S, KU Leuven, Gaston Geenslaan 1, B-3001 Heverlee, Belgium; 3 Department of Biochemistry and Cambridge Systems Biology Center, University of Cambridge, 80, Tennis Court Road, Cambridge, CB2 1GA UK; 4 The Francis Crick Institute, 1 Midland Rd, London, NW11AT UK

## Abstract

Ubiquitin conjugation signals for selective protein degradation by the proteasome. In eukaryotes, ubiquitin is encoded both as a monomeric ubiquitin unit fused to a ribosomal gene and as multiple ubiquitin units in tandem. The polyubiquitin gene is a unique, highly conserved open reading frame composed solely of tandem repeats, yet it is still unclear why cells utilize this unusual gene structure. Using the *Saccharomyces cerevisiae UBI4* gene, we show that this multi-unit structure allows cells to rapidly produce large amounts of ubiquitin needed to respond to sudden stress. The number of ubiquitin units encoded by *UBI4* influences cellular survival and the rate of ubiquitin-proteasome system (UPS)-mediated proteolysis following heat stress. Interestingly, the optimal number of repeats varies under different types of stress indicating that natural variation in repeat numbers may optimize the chance for survival. Our results demonstrate how a variable polycistronic transcript provides an evolutionary alternative for gene copy number variation.

## Abstract (web summary)

Eukaryotic cells rely on the ubiquitin-proteasome system for selective degradation of proteins, a process vital to organismal fitness. Here the authors show that the number of repeats in the polyubiquitin gene is evolutionarily unstable within and between yeast species, and that this variability may tune the cell’s capacity to respond to sudden environmental perturbations.

## Introduction

Timely degradation of proteins by the ubiquitin-proteasome system (UPS) is key to many physiological processes and stress survival^1–4^. Heat stress induces protein misfolding and aggregation, and rapid clearance of misfolded proteins is essential for cells to survive and re-initiate cell division. In eukaryotes, the heat shock (HS) response involves the upregulation of stress-induced genes (e.g., chaperones) and increased ubiquitination of cytosolic proteins^5–7^ thereby increasing the demand for ubiquitin moieties.

Ubiquitin is present in the cell as free and protein-bound ubiquitin pools, which are highly dynamic and interchangeable. Their equilibrium is maintained through tight transcriptional regulation of the various components of the UPS. In eukaryotes, ubiquitin is always encoded by a gene fusion^8, 9^. In yeast, three genes encode ubiquitin as a monomeric ubiquitin unit fused to ribosomal proteins: *RPL40A* (*UBI1*); *RPL40B* (*UBI2*); and *RPS31* (*UBI3*)^8, 10^. One additional ubiquitin gene, *UBI4*, encodes multiple ubiquitin units in tandem^11, 12^. Each of these fusion genes is transcribed as a single transcript and translated into ubiquitin precursors (i.e., a ubiquitin moiety fused to a ribosomal protein or to another ubiquitin) that are subsequently cleaved by specialized proteases called deubiquitinating enzymes (DUBs) to release mature ubiquitin monomers^13^. This process constitutes the *de novo* ubiquitin synthesis. Some DUBs, such as Ubp1, can also cleave unanchored polyubiquitin chains and thus further contribute to the total cellular ubiquitin levels^14^. Ubiquitin monomers can also be regenerated by DUBs from anchored polyubiquitin chains that are released from ubiquitinated proteins before degradation by the proteasome^10^.

As *UBI1*, *UBI2*, and *UBI3* encode ribosomal proteins, their expression correlates with cell growth rate, and is repressed by stress conditions. Conversely, expression of the polyubiquitin *UBI4* gene is stress-inducible and increases after heat stress, upon exposure to DNA-damaging agents, under oxidative stress, in stationary phase and in conditions of zinc deficiency^11, 15–17^. This regulation makes *UBI4*, a gene composed solely of tandem repeats, the main source of *de novo* ubiquitin synthesis under stress conditions.

Tandem repeats are known to be highly unstable as they frequently induce DNA replication slippage or intragenic recombination that lead to the addition or removal of repeat units. Yet many tandem repeats occur within regulatory or coding sequences where their variability can lead to beneficial functional changes^18–20^. The eukaryotic polyubiquitin gene constitutes a special example of a tandem repeat since the entire open reading frame of this gene is composed of several repeats of a DNA sequence that encodes one ubiquitin unit^8^. Despite extensive research on the UPS and its multiple components, the advantages of such a multi-unit gene structure, and whether the (natural) variation in the number of ubiquitin-coding units affects cell physiology have not been previously investigated.

Using the *Saccharomyces cerevisiae* polyubiquitin gene *UBI4* as a model and a combination of genetic and biochemical approaches, we show that the eukaryotic polyubiquitin gene is evolutionarily unstable and that variation in the number of ubiquitin units underlies variation in survival capacity after stress. Interestingly, we found that different *UBI4* repeat numbers are optimal under different stress conditions. We also show that the UPS-mediated degradation kinetics of ubiquitinated proteins during HS depends on the number of ubiquitin repeats. The multiunit structure of the polyubiquitin gene thus constitutes an elegant alternative to gene copy number variation where the number of encoded ubiquitin units allows tuning of the UPS activity, protein homeostasis, and cellular survival during stress.

## Results

### Ubiquitin repeat number varies between and within species

**Fig. 1:**
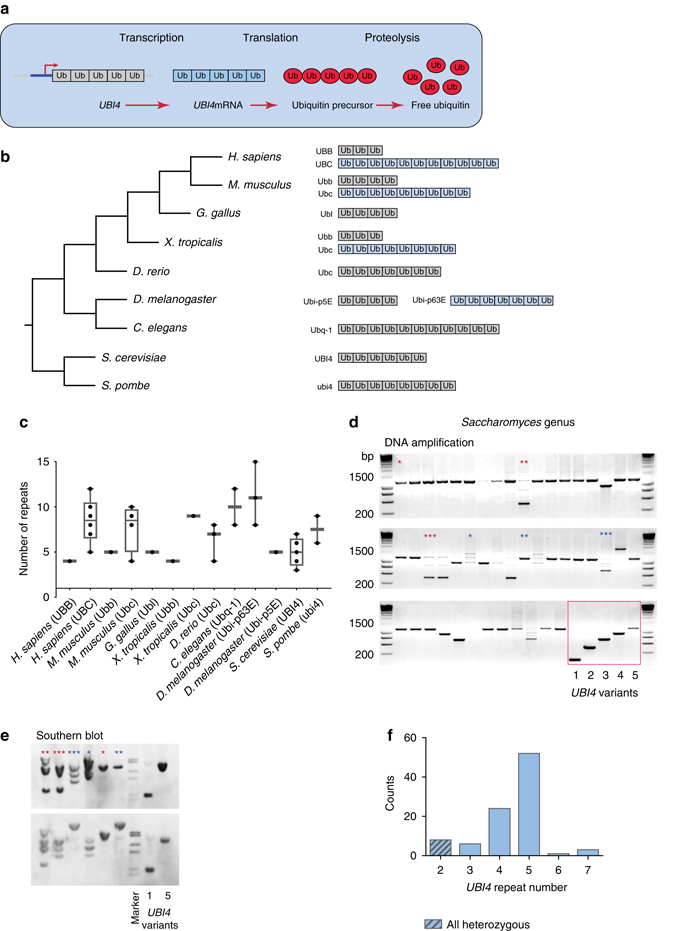
The number of ubiquitin moieties encoded by the eukaryotic polyubiquitin gene is evolutionary unstable and varies among species and strains. **a** The eukaryotic polyubiquitin gene (e.g., *UBI4*) is transcribed as a single transcript and translated into a multiunit ubiquitin precursor that is subsequently cleaved into free ubiquitin moieties by specific deubiquitinating enzymes. **b** Phylogenetic tree of various eukaryotic model organisms (*left*) and their polyubiquitin gene structure(s) (*right*). The variant containing the highest number of ubiquitin repeats is drawn (except for *D. melanogaster*). The gene names for each homologue in the Uniprot database are given. The polyubiquitin gene underwent a duplication event in several eukaryotic lineages (extra gene copy represented in *blue*). In the *D. melanogaster* lineage, the duplicated copies are in tandem. **c** Boxplot depicting the variation in polyubiquitin repeat number for various eukaryotic model organisms (see also **b** and Supplementary Data 1). **d** The polyubiquitin *UBI4* gene was amplified from 98 strains belonging to the *Saccharomyces* genus in their natural ploidy. Shown are 48 representative strains. The framed section shows the amplification products of the different *UBI4* gene variants constructed for this study in the *Saccharomyces cerevisiae* S288c lab strain, with the number under the lanes indicating the number of *UBI4* repeats. *Asterisks* denote the products of DNA amplification and Southern blot (**e**) of the *UBI4* gene originating from the same strains. **e** Southern blot analysis confirms the number of *UBI4* repeats in a subset of the natural strains (**d**), and rules out that the differences in *UBI4* length or the multiple bands shown in **c** are due to slippage during PCR amplification. *Asterisks* denote the products of DNA amplification and Southern blot of the *UBI4* gene originating from the same strains. **f** Distribution of *UBI4* repeat number in the *Saccharomyces* genus. The *UBI4* allele of 2 units is always heterozygous with the other allele being ≥ 4 repeats

The polyubiquitin gene is transcribed and translated into a multiunit ubiquitin precursor that is subsequently cleaved by specific DUBs to yield monomeric ubiquitin moieties^13^ (Fig. 1a). The repetitive nature of the polyubiquitin gene is conserved throughout the eukaryotic lineage^8, 21–24^ (Fig. 1b). We surveyed > 1000 eukaryotic polyubiquitin-coding sequences in the Uniprot database and found extensive variability in the polyubiquitin repeat number between species and strains (Fig. 1c and Supplementary Data 1). In several lineages, including humans and mice, the polyubiquitin gene underwent a duplication event (Fig. 1b) and the resulting paralogs acquired different expression patterns and functional roles^22, 24–27^. A comprehensive genome analysis revealed that one paralog (often the shortest variant) shows little to no changes in repeat number, whereas the longer variant shows extensive repeat number variability (Fig. 1b, c and Supplementary Data 1).

To determine the range of repeat number variability in the polyubiquitin gene within closely related species and strains, we PCR-amplified the *UBI4* gene in 98 natural strains and species of *Saccharomyces* yeasts selected to represent the natural diversity of this genus^28, 29^ (Supplementary Data 2 and Fig. 1d). In the strains for which PCR amplification generated multiple bands, the exact gene size (and hence *UBI4* repeat number) was resolved by Southern blot analysis to rule out slippage events during PCR amplification (Fig. 1e). The *UBI4* allele coding for five units was observed in 53% of the tested strains (Fig. 1f). The results uncover a remarkably high variability in the number of ubiquitin-coding units across the *UBI4* genes in the *Saccharomyces* genus, with some of the diploid or polyploid strains containing *UBI4* alleles of different lengths (Supplementary Data 2). Alleles containing only 2 repeats were always found to be heterozygous, with one allele containing 2 repeats and the other(s) containing at least 4. This suggests that there might be selection against having only a few *UBI4* repeats.

### *UBI4* repeat number influences HS survival

**Fig. 2:**
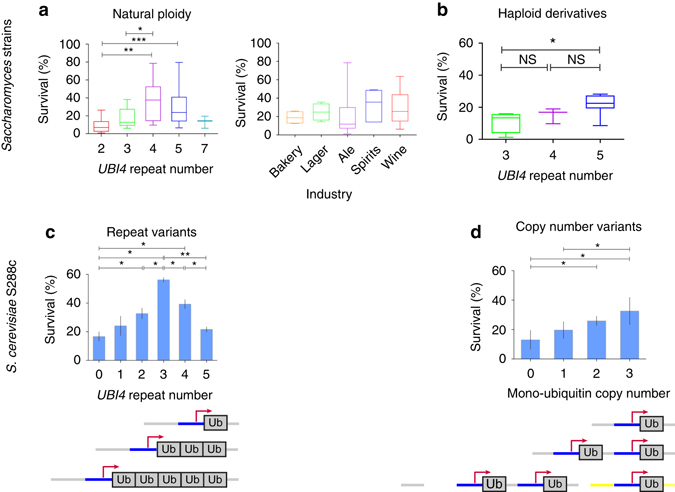
*UBI4* repeat number influences survival after heat shock in natural and engineered *Saccharomyces* strains. **a** Survival of 65 diverse *Saccharomyces* strains after heat shock (43 °C, 4 h) is influenced by the number of ubiquitin units in their *UBI4* gene, and not their industrial niches (right panel). The strains designated as having a *UBI4* gene of 4 or 5 repeats used in this experiment were all homozygous for the *UBI4* gene; strains designated as having 2 or 3 *UBI4* repeats were heterozygous for the *UBI4* gene, with the other allele having 4 or more repeats. **b** Survival levels of haploid derivatives of industrial and feral *S. cerevisiae* strains increase with their *UBI4* repeat number. It should be noted that no haploid strains with a repeat number over 5 were available. **c** Survival after heat shock (44 °C, 4 h) of haploid isogenic *S. cerevisiae* S288c variants containing different numbers of *UBI4* repeats. The scheme shows the *UBI4* gene structure in these repeat variants. Data points represent mean ± SD; *n* = 3. **d** Heat shock survival of isogenic *S. cerevisiae* strains containing multiple copies of a mono-unit *UBI4* gene (i.e., encoding only one ubiquitin unit) correlates with the total number of ubiquitin-coding units. The scheme shows the structure of the mono-ubiquitin copy number variants. The first two copies were inserted in tandem (at the *UBI4* gene locus), whereas the third unit (depicted by the yellow overhangs) was inserted in a neutral genomic location. Data points represent mean ± SD; *n* = 4 or 6. Statistical significance was assessed using the Mann–Whitney test (**a**, **b**) or an unpaired *t*-test (with Welch’s correction) (**c**, **d**). NS, not significant (*P* > 0.05), *P < 0.05, **P < 0.01, ***P < 0.001. For the sake of clarity, only significant differences in survival are shown

To investigate whether the natural variability in *UBI4* repeat number influences cellular fitness in conditions where fast protein turnover is critical, we measured the survival of a set of natural *Saccharomyces* strains after a sudden temperature shift from 30 to 43 °C, a condition known to induce the *UBI4* gene^11, 17^. We find that survival rates under these conditions seem to be affected by the strains’ *UBI4* repeat number, rather than the ecological or industrial niche from which the different yeast strains were isolated (Fig. 2a, *right panel*). Specifically, strains with at least one short *UBI4* allele (2 or 3 units) have significantly lower survival rates than the ones with longer alleles (4 or 5 units; Fig. 2a). Interestingly, strains containing a *UBI4* allele with 7 units display low HS survival indicating that more repeat units do not directly correlate with HS survival (Fig. 2a). It should be noted that out of the 98 natural strains tested, only 3 strains contained a *UBI4* allele with 7 repeats (Fig. 1f), possibly pointing to an unfavorable effect of this allele.

We believe this effect is connected to the ubiquitin repeat number especially given a notable example with 2 genetically related strains (strains Y335 and Y342; Supplementary Data 2 and ref. ^29^). Both strains are used for ale beer production, but have different *UBI4* repeat numbers (2 vs. 4) and show extremely different survival rates during heat stress (15% vs. 79%).

Many industrial and wild strains are polyploid, and can thus carry several alleles of *UBI4* per strain that can each differ in their number of repeats. The presence of multiple alleles of varying repeat length in these polyploids can obscure the effect of *UBI4* repeat number on HS survival. However, we observe a similar, statistically significant difference in survival rates between 14 haploid derivatives of industrial or wild *S*. *cerevisiae* strains. Strains having an allele with 3 units showed a significant reduction in heat stress survival compared to those containing an allele with 5 units (Fig. 2b).

To further disentangle the effect of variation in *UBI4* repeat number on HS survival from the effect of variation in ploidy and genetic background, we generated isogenic variants of the *S. cerevisiae* S288c lab strain that only differ in the number of *UBI4* repeats (Fig. 1d, *framed section*). These strains allowed us to evaluate in a more direct way if differences in the number of ubiquitin-coding units of *UBI4* affect HS survival. After a sudden temperature shift (30 to 44 °C), survival rates first increase with increasing *UBI4* repeat number, reaching a maximum for the variant with 3 repeats before decreasing again (Fig. 2c). These results suggest that the number of ubiquitin moieties synthesized from the *UBI4* gene directly influences cell survival following a sudden HS.

To rule out ubiquitin-independent effects responsible for the fitness differences, we created a one-repeat *UBI4* variant that contains a Lys48Arg mutation that prevents this moiety from being used in polyubiquitination reactions. This mutant has a survival rate comparable to the mutant with a complete *UBI4* deletion and significantly lower than the strain with an intact one-repeat *UBI4* allele (Supplementary Fig. 1). This indicates that the survival rates after HS are directly dependent on ubiquitin produced from the *UBI4* gene.

To determine if our observed phenotypes were in fact a dose-dependent consequence of multiple units, we created mono-ubiquitin copy number variants. In these variants, we replaced the *UBI4* gene by one or two copies (in tandem) of a single ubiquitin moiety, each controlled by its own promoter (Fig. 2d). A third ubiquitin moiety was inserted at a neutral genomic location (depicted by the yellow overhangs in Fig. 2d) to obtain a variant with 3 copies of a single ubiquitin unit (Fig. 2d). The survival of these variants after a sudden HS also increases with the number of mono-ubiquitin moieties (Fig. 2d), similar to what we observe with the tandem repeat number variants (Fig. 2c). These results suggest that having multiple ubiquitin-encoding repeats within one gene or multiple copies of a mono-ubiquitin-coding gene may increase the cells’ capacity to generate the large amounts of ubiquitin needed for the proteome to quickly adapt to the HS. Our data also suggest that having multiple copies of a mono-ubiquitin-encoding gene can (partially) recapitulate the HS survival phenotype of a strain that has a multiunit gene structure (polyubiquitin). However, it should be noted that HS survival of strains with a multiunit gene structure is often slightly better compared to strains encoding multiple copies of mono-ubiquitin.

### Optimal *UBI4* repeat number is condition-specific

Considering the variability in natural *UBI4* repeat number, we further investigated the possible role of *UBI4* repeat number variation in surviving other types of stress, using the isogenic *UBI4* repeat variants (Fig. 2c). Oxidative stress and nitrogen starvation have both been linked to *UBI4*, as *ubi4* null mutants display increased sensitivity to both types of stress^15^. When the isogenic *UBI4* repeat number variants were grown on a nitrogen-starved medium at an elevated temperature, the short variants (≤2 repeats) showed reduced fitness relative to the long variants (≥3 repeats; Supplementary Fig. 2). Interestingly, under these conditions, the *UBI4* allele encoding 5 repeats gives the clearest growth advantage.

A similar trend was seen when cells were exposed to oxidative stress. While all strains displayed a reduced maximal growth rate and increased recovery (lag) time under oxidative stress conditions compared to no stress conditions (YPD, Yeast extract, Peptone and Dextrose) (Supplementary Fig. 3), strains containing 5 *UBI4* repeats reproducibly grew better under oxidative stress compared to *UBI4*-null or single-repeat strains (higher growth rate in the presence of 2.5 mM paraquat; unpaired *t*-test, two-sided *p*-values < 0.05). Although 3 repeats appeared optimal for HS recovery, under oxidative stress these strains took significantly longer to recover than single-repeat strains (longer lag time; unpaired *t*-test, two-sided *p*-value = 0.0461).

A recent study showed that a 2-repeat *UBI4* variant is less fit in zinc-deficient conditions relative to a 5-repeat variant^16^. In line with our findings, a recent large-scale study also showed that the exact expression level of a gene required for optimal fitness is highly condition-specific^30^. Taken together with our results, the variation in the “best” repeat number demonstrates that there is no single number of repeats in *UBI4* that is optimal in all possible environments. Rather, variation in *UBI4* repeat number is likely an evolutionary “tuning knob” that allows strains to optimize their stress-induced ubiquitin system, with different repeat numbers being optimal under different stress conditions (type, severity, and duration).

### *UBI4* is main source of de novo ubiquitin during heat stress

**Fig. 3:**
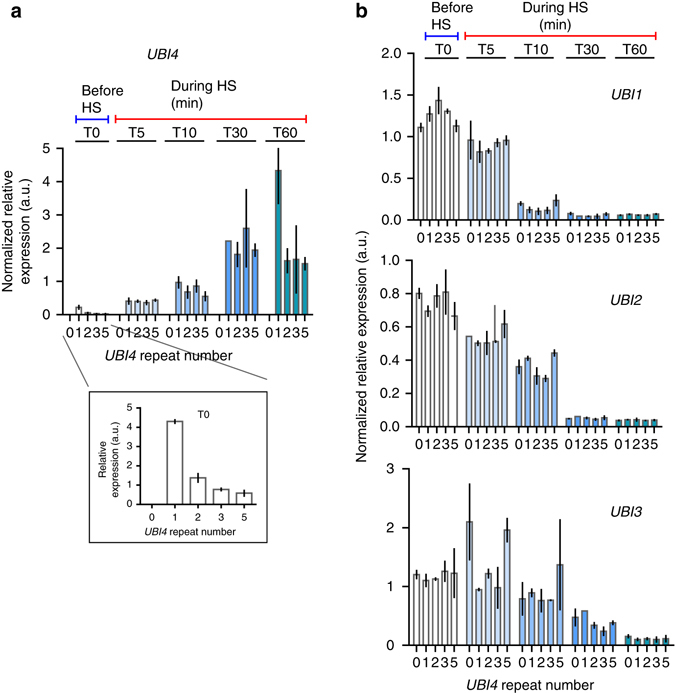
Time course of expression of all ubiquitin-coding genes in the *UBI4* repeat variants during heat shock. **a** Time series of *UBI4* expression in the repeat variants (carrying a *UBI4* gene encoding either 0, 1, 2, 3, or 5 ubiquitin units) during a sustained heat shock (HS) at 44 °C. *UBI4* transcripts were measured by real-time quantitative PCR (RT-qPCR) using primers that specifically anneal to one ubiquitin moiety allowing the measurement of the transcripts from each *UBI4* repeat variant rather than the total number of ubiquitin moieties per transcript. *Inset* shows the expression of *UBI4* at time point 0 (before HS) in the repeat variants. **b** Time series of *UBI1*, *UBI2*, and *UBI3* expression in the *UBI4* repeat variants during the HS at 44 °C. Primers specific for the ribosomal protein-coding region were used to distinguish between the different genes. **a**, **b** Data are mean of 2 biological replicates (and 2 technical replicates per sample) ± SD

In *S. cerevisiae*, the majority of ubiquitin is provided by the monomeric *UBI1* (*RPL40A*), *UBI2* (*RPL40B*), and *UBI3* (*RPS31*) genes under physiological growth conditions, whereas the polyubiquitin gene *UBI4* has been shown to be the major source of ubiquitin during stress^11, 17^. To check if differences in transcription of the *UBI4* gene could explain the differences in survival observed for the different *UBI4* repeat number variants (Fig. 2), we used real-time quantitative PCR (RT-qPCR; see Supplementary Information for more details on specific primer design—we measured the number of transcripts from each *UBI4* repeat variant, rather than the total number of ubiquitin moieties). We monitored the transcriptional response of all ubiquitin-coding genes in the *UBI4* repeat number variants during a time course after the cells were shifted from 30 to 44 °C. We observe that during this sustained HS, *UBI4* induction is quick and transient, reaching a maximum after 30 min, confirming previous reports^17^ (Fig. 3a). *UBI4* transcripts in all the variants increase to a similar level after the cells are shifted to a higher temperature, except for the 1-repeat variant whose induction continues to increase after 30 min (Fig. 3a). Surprisingly, the steady-state expression of *UBI4* before the heat stress (at 30 °C) differs between the repeat variants and reveals an anticorrelation between the *UBI4* mRNA levels and its number of ubiquitin repeats (Fig. 3a, *inset*). For example, *UBI4* transcripts are ~ 7-fold higher in the 1-repeat variant and ~ 2.5-fold higher in the 2-repeat variant relative to the wild type (5 repeats). These observations suggest that a basal level expression of *UBI4* gene is necessary for maintaining adequate ubiquitin pools in non-stressed conditions and therefore *UBI4* likely also plays a role in physiological protein homeostasis in *S. cerevisiae*, with cells that contain fewer *UBI4* repeats compensating for the lack of ubiquitin by increasing basal *UBI4* expression.

The ribosomal-fusion *UBI1*, *UBI2*, and *UBI3* genes are downregulated during HS and their expression follows the opposite trend compared to *UBI4* (Fig. 3b). This agrees with previous reports showing that ribosomal genes are downregulated during stress^31^. Importantly, we find no significant differences in the ribosomal-fusion ubiquitin transcripts (*UBI1-3*) between the *UBI4* repeat variants (Fig. 3b). Taken together, these results confirm that during stress conditions, the bulk of ubiquitin is provided by *UBI4* with no or very little contribution from *UBI1*-*3*. Moreover, our data suggest that the main contribution to differences in HS survival of the *UBI4* variants arises from differences in the amount of ubiquitin moieties generated from the *UBI4* gene (see also below).

### *UBI4* repeat number affects UPS activity during heat stress

Although there are no significant differences in the number of mRNA ubiquitin transcripts in the *UBI4* variants with 2, 3, or 5 repeats during HS (Fig. 3), these transcripts are of different length and encode different numbers of ubiquitin moieties. Therefore, part of the difference in HS survival between these *UBI4* repeat variants could potentially be explained by differences in the overall number of ubiquitin moieties present in the cell. To examine the effects of differential ubiquitin production from the *UBI4* gene on global ubiquitination levels, we extracted soluble proteins from the *UBI4* variants at multiple time points during a HS and measured total ubiquitination by western blot (Supplementary Fig. 4). This type of analysis is qualitative and only has the power to resolve large differences in total polyubiquitination yet we clearly observe less polyubiquitination of soluble proteins in the *UBI4* variant with 0 and 1 repeat relative to the higher repeat variants. Polyubiquitination levels at a physiological temperature were comparable across all *UBI4* variants (HS 0 min) and after 30 min of HS, there is clearly more polyubiquitin in strains with ≥2 repeats. Since this method cannot resolve minor differences, we cannot exclude the possibility of variation in polyubiquitination within the higher (2–5) repeat variants.

**Fig. 4:**
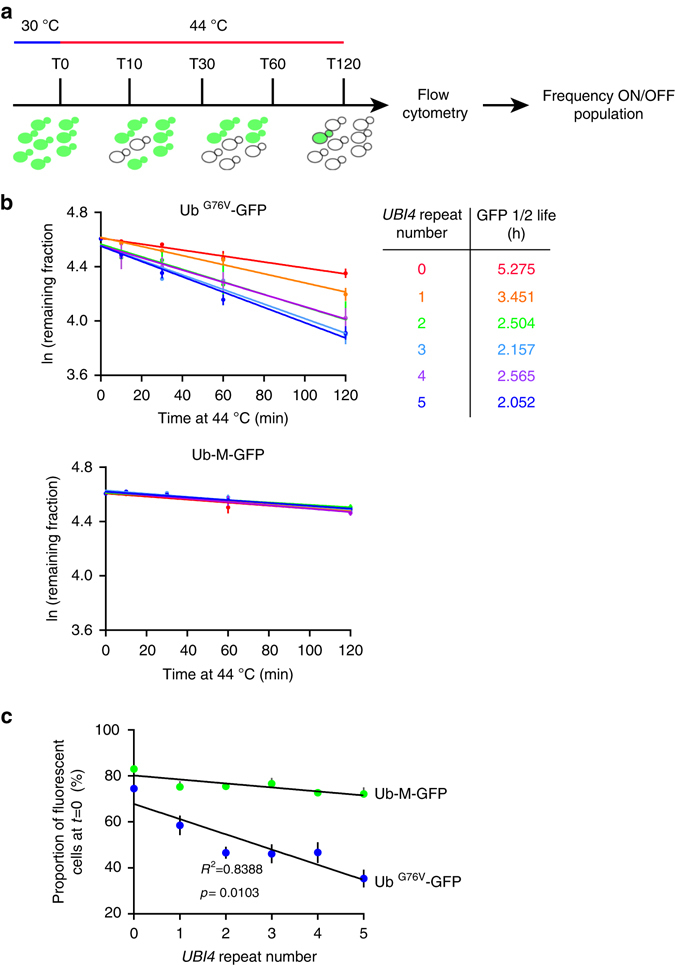
Quantitative protein turnover measurements during heat shock reveal *UBI4* repeat number-dependent degradation kinetics. **a** Experimental design for turnover measurements using fluorescent protein reporters. *UBI4* repeat variants expressing Ub^G76V^-GFP (a substrate for UPS-mediated proteolysis) or Ub-M-GFP (stable GFP)^27, 28^ were used to measure the UPS activity during heat shock (44 °C). Cells were grown at 30 °C until exponential phase in SC medium with 2% galactose to induce the expression of the fluorescent reporters. The cultures were then shifted to 44 °C and samples were taken before the temperature shift (T0) and at multiple time points after for flow cytometry analysis. For each time point, the fraction of fluorescent cells over the total cell population was calculated. **b** We then fit the fraction to a first-order decay function to calculate the degradation rate constant *K*_deg_. Since growth is completely arrested during the heat shock period, we do not expect any contribution from protein dilution rates (as a result of cell division). Protein half-lives were calculated using the formula *T*_(1/2)_ = ln (2)/*K*_deg_. **c** The initial proportion (i.e., before heat shock) of fluorescent cells over the total population gives the steady-state levels of the reporter proteins and reflects the UPS activity under physiological conditions. **b**, **c** Data are mean of 4 independent replicates ± SEM

Proteasomal degradation dynamics can be influenced by several factors, including the configuration of the ubiquitin chains on the protein substrate as well as the total number of ubiquitin moieties present on the substrate^32, 33^. To unravel possible differences in the UPS activity between *UBI4* repeat variants and check whether lower polyubiquitination in the short *UBI4* variants results in slower substrate degradation, we employed a well-established, quantitative assay to measure UPS-dependent protein degradation in single cells using a modified green fluorescent protein (GFP)^34, 35^. We measured fluorescence in *UBI4* repeat variants expressing Ub^G76V^-GFP, a substrate for ubiquitin-proteasome-dependent proteolysis, or the stable Ub-M-GFP as a control, after a temperature shift from 30 to 44 °C (Fig. 4a). We determined the turnover rates of ubiquitinated GFP, and by extension the UPS activity, by calculating the frequency of the remaining fluorescent cells over the total number of cells at multiple time points during the HS (Fig. 4a and Methods for details). Whereas Ub^G76V^-GFP was degraded with a half-life of ~ 2 h in cells containing a 5-repeat *UBI4* during HS, its half-life gradually became longer with decreasing *UBI4* repeat number (Fig. 4b). In contrast, the levels of the stable form of GFP (Ub-M-GFP) were the same for all strains and were relatively unchanged throughout the course of the HS (Fig. 4b, *lower panel*).

To ensure that the observed differences in half-life were not due to differences in substrate (GFP) expression, we measured the levels of GFP-mRNA by RT-qPCR. mRNA levels remain virtually unchanged during the HS experiment, and are also similar across all the *UBI4* repeat variants (Supplementary Fig. 6 and Supplementary Data 4). Moreover, degradation of Ub^G76V^-GFP was drastically slowed down upon proteasome inhibition by addition of MG132 (Supplementary Fig. 7), further indicating that the degradation of Ub^G76V^-GFP takes place via the proteasome. It is also interesting to note that the strain with the fastest protein turnover (5-repeat *UBI4*) does not display the best survival after sudden HS (Fig. 2c). This underscores that faster protein turnover is not necessarily better for cellular fitness (see also Discussion).

Interestingly, the steady-state levels of Ub^G76V^-GFP at a physiological temperature (30 °C) negatively correlated with the number of *UBI4* repeats (Fig. 4c). Since growth profiles at 30 °C are comparable between the *UBI4* variants (Supplementary Fig. 8), we do not expect any noticeable contribution of protein dilution. The differences at this physiological temperature therefore likely result from variation in UPS-mediated proteolysis, with variation in *UBI4* repeat number potentially affecting proteasome activity even at physiological temperatures.

## Discussion

Many studies have highlighted the critical roles of ubiquitin homeostasis for normal cellular physiology and stress response. In mice, the failure to recycle ubiquitin from proteasomal substrates results in synaptic dysfunction^36^ and deletion of either polyubiquitin gene paralog underlies severe pathological phenotypes^26, 27^. Additionally, alteration in protein half-life can result in developmental abnormalities, neurodegeneration, and cancer^37^. In yeast, depletion of the free mono-ubiquitin pool renders the cells susceptible to translational inhibition^38^. But why would cells need a polycistronic ubiquitin gene to survive stressful conditions? What are the advantages of a multiunit gene structure? Does the natural variability in the number of ubiquitin-coding units affect cell physiology and stress responses?

Using a combination of genetic and biochemical approaches, we sought to address this gap in our understanding of ubiquitin biology. We show that the variation in the number of ubiquitin repeats encoded by the polyubiquitin gene (*UBI4*) underlies variation in stress resistance. The *UBI4* variants with short repeat number (≤ 2 repeats) show increased sensitivity to a wide range of stresses, whereas the longer repeat alleles provide increased resistance to stress. Interestingly, this effect on stress resistance is highly condition-specific, indicating that certain alleles are optimal for fitness in one stress condition, but not in another.

Our results also indicate that repeat variation in the polyubiquitin gene can tune the degradation kinetics of ubiquitinated proteins during stress. Short *UBI4* repeat variants display a very slow turnover of ubiquitinated substrates. Gradual increases in the number of *UBI4* units result in faster proteolysis of ubiquitinated substrates (Fig. 4b). In other words, the activity of the UPS in stress conditions, and consequently the clearance of misfolded proteins, can be tuned by the amount of ubiquitin produced from the polyubiquitin gene. Our study thus adds another factor that can underlie variation in the activity of the UPS during stress—namely variation in the polyubiquitin gene repeat number—in addition to other factors recently described^39^.

The natural variation in the number of ubiquitin moieties encoded by the polyubiquitin gene could thus affect the proteome half-life dynamics in living cells under stress (e.g., heat, starvation, and oxidative stress) and subsequently play a role in stress resistance and survival. Our data also suggest that variation in the number of *UBI4* ubiquitin-coding units can affect proteostasis under non-stress conditions (Fig. 4c).

In sum, our study takes an in-depth look the physiological effects of changes in unit number of the polyubiquitin gene. Our results thus provide a distinct example of how a eukaryotic transcript that contains a tandem repeat of a coding unit serves as an elegant alternative for gene copy number variation, enables cells to quickly produce more copies of a specific open reading frame. Moreover, given the frequent replication slippage or intragenic recombination events that occur at tandem repeat sites (events that are less frequent if the copies would be encoded at different locations spread across the genome), it seems plausible that such a repetitive gene is highly variable and “evolvable”, providing cells with an opportunity to quickly evolve novel stress-induced polyubiquitin gene alleles that result in optimal tuning of internal ubiquitin levels in changing conditions or niches.

## Methods

### Strain construction

Details of the strategy used to create the *S. cerevisiae UBI4* repeat variants can be found in Supplementary Information. A full list of primers and strains are provided in Supplementary Data 2 and 3.

### HS survival assays

To quantify survival rates after HS, we grew the cultures first in YPD at 30 °C till OD_600_ = 3–4, diluted them to OD_600_ = 0.1 in SC (Synthetic Complete) medium containing 2% glucose and transferred 50 μl to a thermocycler for the HS period (4 h). Using the gradient function of the thermocycler we could induce a rapid shift from a physiological growth temperature (30 °C) to different heat stress temperatures (38 to 45 °C) in parallel on multiple samples. We calculated the survival rates (at a given temperature) by dividing the number of colony-forming units obtained for each sample before and after the temperature switch.

### Southern blotting

Southern blotting was performed using a standard protocol^40^ on 10 μg of gDNA isolated using the Genomic tip 100/G kit (Qiagen). The blots of NdeI-/EcoRI-digested gDNA were hybridized with a digoxigenin (DIG)-labeled probe prepared by PCR amplification of a *UBI4* coded ubiquitin moiety using primers 4028–4029, gDNA from RG525, and the PCR DIG Probe Synthesis kit (Roche).

### Growth assays

For nitrogen starvation assays, overnight cultures grown in YPD were normalized to OD_600_ = 1 in water and 10-fold serially diluted. Ten microliters of each dilution were spotted onto synthetic low-ammonia dextrose plates (nitrogen-poor medium) and incubated at 37 or 41 °C.

For oxidative stress assays, *UBI4* repeat variants were grown in YPD to OD_600_ = 0.1. These cultures were used to inoculate fresh YPD in the presence and absence of 2.5 mM paraquat (Sigma Aldrich). Growth was monitored at 600 nm using a Bioscreen C (Growth Curves, USA) maintained at 30 °C with continuous shaking for 30 h. Maximal growth rate and lag time were calculated in accordance with Swinnen *et al*.^41^ using an in-house-built script in R similar to that described by New *et al*.^42^. Briefly, lag times were calculated from the growth data using R. First, the optical density (OD) values were subtracted by the background values of wells containing no culture, and the resulting observed ODs (OD_obs_) were corrected to account for the nonlinearity between cell density (OD_cor_) and observed OD at higher cell densities (using the formula OD_cor_ = OD_obs_ + 0.449(OD_obs_)^2^ + 0.191(OD_obs_)^3^ as in ref. ^43^). Next, the growth rates were calculated by taking the discrete first derivate of the natural logarithm of OD_cor_ vs. time, and smoothening this using a smoothing spline (R function smooth.spline with spar = 0.2). From these data, the maximum growth rate was calculated as the average of the five highest values. The lag time could then be calculated as the time corresponding to the intersection point between two lines on an OD vs. time graph. The first line is an exponential curve that is extrapolated from the point of maximum growth rate and the second line is an horizontal line crossing the vertical axis at the initial OD^41^.

For growth assays in glucose, *UBI4* repeat variants were grown overnight in YPD. The next day, these cultures were used to inoculate fresh YPD. Growth was monitored at 600 nm using a Bioscreen C (Growth Curves) maintained at 30 °C with continuous shaking.

### Real-time quantitative PCR

To monitor the expression of all ubiquitin-coding genes during HS, we grew biological duplicates of the *UBI4* repeat variants overnight till saturation in YPD. We then diluted the cultures to OD_600_ = 0.1 in 50 ml SC medium containing 2% glucose and grew them at 30 °C till OD_600_ = 0.5. We then transferred the cultures to a water bath at 44 °C and at 0, 5, 10, 30, and 60 min after the temperature shift we mixed 6 ml cultures with an equal volume of ice-cold water and collected the cells by centrifugation. We extracted total RNA using the standard phenol–chloroform method^44^ and synthesized cDNA from 1 μg total RNA using the QuantiTect Reverse Transcription kit (Qiagen). RT-qPCR was performed using the StepOnePlus system (Applied Biosystems). To design RT-qPCR primers that allow us to measure the transcripts from each *UBI4* repeat variant, rather than the total number of ubiquitin moieties per transcript, we chose primers that specifically anneal to one ubiquitin moiety by taking advantage of the polymorphisms present between the various ubiquitin moieties in the *UBI4*-coding sequence. We verified the specificity of the primers by using various concentrations of gDNA from each *UBI4* variant. We obtained C_T_ values that were proportional to the concentration of gDNA used and were similar for the *UBI4* repeat variants. *UBI4* expression was normalized to the expression of the *ACT1* gene. To account for differences between RT-qPCR runs and in order to compare trends between different time points, we first normalized to the C_T_ signal from the same amount of gDNA present in each run. We then calculated the ratio of the normalized values to obtain the relative expression levels. Primers used for RT-qPCR are listed below.

### Western blotting

To assess polyubiquitination levels in the *UBI4* repeat variants during HS, overnight-saturated cultures were diluted in 50 ml YPD and grown at 30 °C till OD_600_ = 0.6–0.8. Before HS, we harvested cells from 10 ml cultures and transferred the remaining cultures to a water bath at 44 °C. At 15, 30, and 60 min after temperature switch, we again harvested cells from 10 ml cultures. Cell pellets were lysed with glass beads in cold lysis buffer (50 mM Tris (pH 7.4), 100 mM NaCl, 1 mM EDTA, 0.1% Triton X-100, and protease inhibitor cocktail (Roche)) in a FastPrep-24 homogenizer (MP Biomedicals). Extracts were cleared by centrifugation (10 min, 14,000 r.p.m. at 4 °C) and protein concentrations were measured by the Bradford assay (Protein Quantification kit, Sigma-Aldrich). Total proteins (5 μg) were separated by 10–20% gradient gels (Life Technologies) using a Tricine-based buffer, followed by transfer to polyvinylidene difluoride membranes. Blots were incubated with mouse anti-ubiquitin monoclonal antibody MAB1510 (Millipore, 1:2000) followed by incubation with rabbit anti-mouse IgG-horseradish peroxidase-conjugated antibody (ab97046, Abcam, 1:20,000), and western signals were detected using the LumiGLO Ultra Chemiluminescence kit (KPL).

### Determination of UPS activity during HS

*UBI4* repeat variants carrying plasmids pYES-Ub-M-GFP (stable GFP) or pYES-Ub^G76V^-GFP (ubiquitin fusion degradation substrate)^27, 28^ (Addgene) were grown for two successive overnights at 30 °C in SC-URA medium containing 2% glucose and then 2% raffinose and 2% galactose. The cultures were next diluted in SC-URA medium containing 2% galactose and grown at 30 °C until midlog phase and then transferred to a water bath at 44 °C. At 0, 10, 30, 60, and 120 min after the temperature shift, 100 µl cultures were centrifuged and cells frozen in 25% glycerol until flow cytometry analysis. Fluorescence of 50,000 cells per sample was measured using a BD Influx flow cytometer equipped with a 488 nm laser and a 530/40 nm filter (GFP). We calculated the frequency of the remaining fluorescent cells over the total population at various time points using the FlowJo software (Tree Star, Inc.). Details on the calculation of the turnover rates are provided in Supplementary Information. A similar setup was used to check effect of proteasomal inhibition, where MG-132 (Sigma) was added to a final concentration of 100 µM.

### Determination of Ub^G76V^-GFP turnover rates

To calculate the degradation rates of Ub^G76V^-GFP, we first normalized the remaining fluorescence of each sample at a given time point relative to its initial fluorescence before HS (assigned an arbitrary value of 1). The normalized data were log-transformed and fitted to a straight line using GraphPad Prism. To evaluate the quality of curve fitting, we calculated the goodness of fit of linear regression (*R* ^2^) and obtained *R* ^2^ > 0.9 in all cases. The average decay rate constant *k* (i.e., slope) of the best-fit curve from 4 independent measurements was determined and used to calculate the half-life (*T*_1/2_) of GFP as follows: ${T_{1/2}} = \frac{{{\rm{ln}}\left( 2 \right)}}{k}$

### GFP expression analysis

Cultures were grown essentially as described in ‘Determination of UPS activity during HS.’

Before being subjected to HS, 6 ml cultures were taken as T0 samples, 6 ml cold diethyl pyrocarbonate-treated water was added to each sample. Cell pellets were immediately collected and stored at −80 °C for later RNA isolation. The same sample-taking procedure was performed at different time points during HS.

RNA was isolated using the MasterPure Yeast RNA Purification Kit. The expression levels of GFP were analyzed by RT-PCR using primers PYY144 and PYY145, and the expression levels of *ACT1* were analyzed using primers PYY140 and PYY141. A delta-delta Ct method was used (with T0 being the control condition and *ACT1* being the control gene) to normalize GFP expression levels. Primers are listed in Supplementary Data 3.

### Data availability

The data supporting the findings of this study are available from the corresponding author upon request.

## Electronic supplementary material

Supplementary Information

Supplementary Data 1

Supplementary Data 2

Supplementary Data 3

Supplementary Data 4

## References

[1] Finley D (2009). Recognition and processing of ubiquitin-protein conjugates by the proteasome. Annu. Rev. Biochem..

[2] Goldberg AL (2003). Protein degradation and protection against misfolded or damaged proteins. Nature.

[3] Hershko A, Ciechanover A (1998). The ubiquitin system. Annu. Rev. Biochem..

[4] Hochstrasser M (1996). Ubiquitin-dependent protein degradation. Annu. Rev. Genet..

[5] Fang NN (2014). Rsp5/Nedd4 is the main ubiquitin ligase that targets cytosolic misfolded proteins following heat stress. Nat. Cell Biol..

[6] Fang NN (2011). Hul5 HECT ubiquitin ligase plays a major role in the ubiquitylation and turnover of cytosolic misfolded proteins. Nat. Cell Biol..

[7] Parag HA, Raboy B, Kulka RG (1987). Effect of heat shock on protein degradation in mammalian cells: involvement of the ubiquitin system. EMBO J..

[8] Ozkaynak E (1987). The yeast ubiquitin genes: a family of natural gene fusions. EMBO J..

[9] Wiborg O (1985). The human ubiquitin multigene family: some genes contain multiple directly repeated ubiquitin coding sequences. EMBO J..

[10] Finley D, Bartel B, Varshavsky A (1989). The tails of ubiquitin precursors are ribosomal proteins whose fusion to ubiquitin facilitates ribosome biogenesis. Nature.

[11] Finley D, Ozkaynak E, Varshavsky A (1987). The yeast polyubiquitin gene is essential for resistance to high temperatures, starvation, and other stresses. Cell.

[12] Ozkaynak E, Finley D, Varshavsky A (1984). The yeast ubiquitin gene: head-to-tail repeats encoding a polyubiquitin precursor protein. Nature.

[13] Reyes-Turcu FE, Ventii KH, Wilkinson KD (2009). Regulation and cellular roles of ubiquitin-specific deubiquitinating enzymes. Annu. Rev. Biochem..

[14] Tobias JW, Varshavsky A (1991). Cloning and functional analysis of the ubiquitin-specific protease gene UBP1 of *Saccharomyces cerevisiae*. J. Biol. Chem..

[15] Cheng L, Watt R, Piper PW (1994). Polyubiquitin gene expression contributes to oxidative stress resistance in respiratory yeast (*Saccharomyces cerevisiae*). Mol. Gen. Genet..

[16] MacDiarmid CW (2016). Activation of the yeast UBI4 polyubiquitin gene by Zap1 transcription factor via an intragenic promoter is critical for zinc-deficient growth. J. Biol. Chem..

[17] Simon JR, Treger JM, McEntee K (1999). Multiple independent regulatory pathways control UBI4 expression after heat shock in Saccharomyces cerevisiae. Mol. Microbiol..

[18] Gemayel R (2015). Variable glutamine-rich repeats modulate transcription factor activity. Mol. Cell.

[19] Gemayel R (2010). Variable tandem repeats accelerate evolution of coding and regulatory sequences. Annu. Rev. Genet..

[20] Vinces MD (2009). Unstable tandem repeats in promoters confer transcriptional evolvability. Science.

[21] Callis J (1995). Structure and evolution of genes encoding polyubiquitin and ubiquitin-like proteins in *Arabidopsis thaliana* ecotype Columbia. Genetics.

[22] Radici L (2013). Ubiquitin C gene: structure, function, and transcriptional regulation. Sci. Res..

[23] Tachikui H (2003). Lineage-specific homogenization of the polyubiquitin gene among human and great apes. J. Mol. Evol..

[24] Zhan Z (2012). Rapid functional divergence of a newly evolved polyubiquitin gene in *Drosophila* and its role in the trade-off between male fecundity and lifespan. Mol. Biol. Evol..

[25] Lu C, Kim J, Fuller MT (2013). The polyubiquitin gene Ubi-p63E is essential for male meiotic cell cycle progression and germ cell differentiation in *Drosophila*. Development.

[26] Ryu K-Y (2007). The mouse polyubiquitin gene UbC is essential for fetal liver development, cell-cycle progression and stress tolerance. EMBO J..

[27] Ryu K-Y (2008). The mouse polyubiquitin gene Ubb is essential for meiotic progression. Mol. Cell. Biol..

[28] Gallone B (2016). Domestication and divergence of saccharomyces cerevisiae beer yeasts. Cell.

[29] Steensels J (2014). Large-scale selection and breeding to generate industrial yeasts with superior aroma production. Appl. Environ. Microbiol..

[30] Keren L (2016). Massively parallel interrogation of the effects of gene expression levels on fitness. Cell.

[31] Gasch AP (2000). Genomic expression programs in the response of yeast cells to environmental changes. Mol. Biol. Cell.

[32] Lu Y (2015). Substrate degradation by the proteasome: a single-molecule kinetic analysis. Science.

[33] Thrower JS (2000). Recognition of the polyubiquitin proteolytic signal. EMBO J..

[34] Dantuma NP (2000). Short-lived green fluorescent proteins for quantifying ubiquitin/proteasome-dependent proteolysis in living cells. Nat. Biotechnol...

[35] Heessen S (2003). Inhibition of ubiquitin/proteasome-dependent proteolysis in *Saccharomyces cerevisiae* by a Gly-Ala repeat. FEBS Lett..

[36] Chen P-C (2011). Ubiquitin homeostasis is critical for synaptic development and function. J. Neurosci..

[37] Ciechanover A (2012). Intracellular protein degradation: from a vague idea through the lysosome and the ubiquitin-proteasome system and onto human diseases and drug targeting. Neuro-degen. Dis..

[38] Hanna J, Leggett DS, Finley D (2003). Ubiquitin depletion as a key mediator of toxicity by translational inhibitors. Mol. Cell. Biol..

[39] Fang NN (2016). Deubiquitinase activity is required for the proteasomal degradation of misfolded cytosolic proteins upon heat-stress. Nat. Commun..

[40] Green, M. R. & Sambrook J. *Molecular Cloning: A Laboratory Manual* (Cold Spring Harbor Laboratory Press, 2012).

[41] Swinnen IA (2004). Predictive modelling of the microbial lag phase: a review. Int. J. Food Microbiol..

[42] New AM (2014). Different levels of catabolite repression optimize growth in stable and variable environments. PLoS Biol..

[43] Warringer J, Blomberg A (2003). Automated screening in environmental arrays allows analysis of quantitative phenotypic profiles in *Saccharomyces cerevisiae*. Yeast.

[44] Amberg, D., Burke D. & Strathern J. *Methods in Yeast Genetics: A Cold Spring Harbor Laboratory Course Manual* (Cold Spring Harbor Laboratory Press, 2005).

