# Supplementary material for: Variable repeats in the eukaryotic polyubiquitin gene ubi4 modulate proteostasis and stress survival
Source: Nat Commun. 2017 Aug 30;8:397. doi: 10.1038/s41467-017-00533-4 (PMC5577197; doi:10.1038/s41467-017-00533-4)
Supplement: Supplementary file 1 — Supplementary Information [file 41467_2017_533_MOESM1_ESM.pdf]

File Name: Supplementary Information
Description: Supplementary Figures, Supplementary Methods and Supplementary References

File Name: Supplementary Dataset 1: Analysis of the variability in the number of ubiquitin-coding repeats in the polyubiquitin gene across the eukaryotic kingdom
Description: We extracted the polyubiquitin encoding transcripts from all available eukaryotic sequences in the Uniprot database. The first tab shows the distribution of the polyubiquitin repeat number in the eukaryotic kingdom. The second tab shows the Uniprot ID and the repeat numbers identified for each homologue. The third tab shows the polyubiquitin orthologs in the nine model organisms shown in Fig. 1b. This dataset is provided as a separate Excel file.

File Name: Supplementary Dataset 2: List of natural and industrial Saccharomyces strains used in this study.
Description: Table containing strain name, origin (industry) and number of repeats within the UBI4 gene of natural and industrial Saccharomyces strains, as well as a list of all Saccharomyces cerevisiae strains created in this study. This dataset is provided as a separate Excel file.

File Name: Supplementary Dataset 3
Description: List of primers used in this study.

File Name: Supplementary Dataset 4: Statistical analyses of data depicted in Supplementary Figure 6.
Description: P-values (Tukey HSD test) for data shown in Supplementary Fig. 6 are given. This datset is provided as a separate Excel file.

Supplementary Data

Supplementary Figure 1

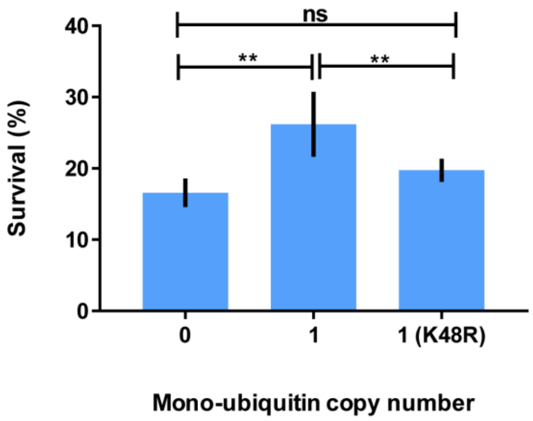

Supplementary Figure 1. Loss of *UBI4*-encoded ubiquitin for (poly)ubiquitination reactions results in decreased heat shock survival

Heat shock survival (44°C – 4h) of isogenic *S. cerevisiae* S288c variants containing a *UBI4* gene encoding either 0 or 1 ubiquitin unit. Mutating residue Lys48 to Arg (thus preventing this moiety from being used in polyubiquitination reactions) in this single ubiquitin unit-encoding *UBI4* gene significantly reduces its heat shock survival. Statistical significance was assessed using Tukey multiple comparisons of mean. ns, not significant ($p>0.05$), * $p<0.05$, ** $p<0.01$, *** $p<0.001$.

Data are mean of 4 independent biological replicates. Data points represent mean ± SD.

Supplementary Figure 2

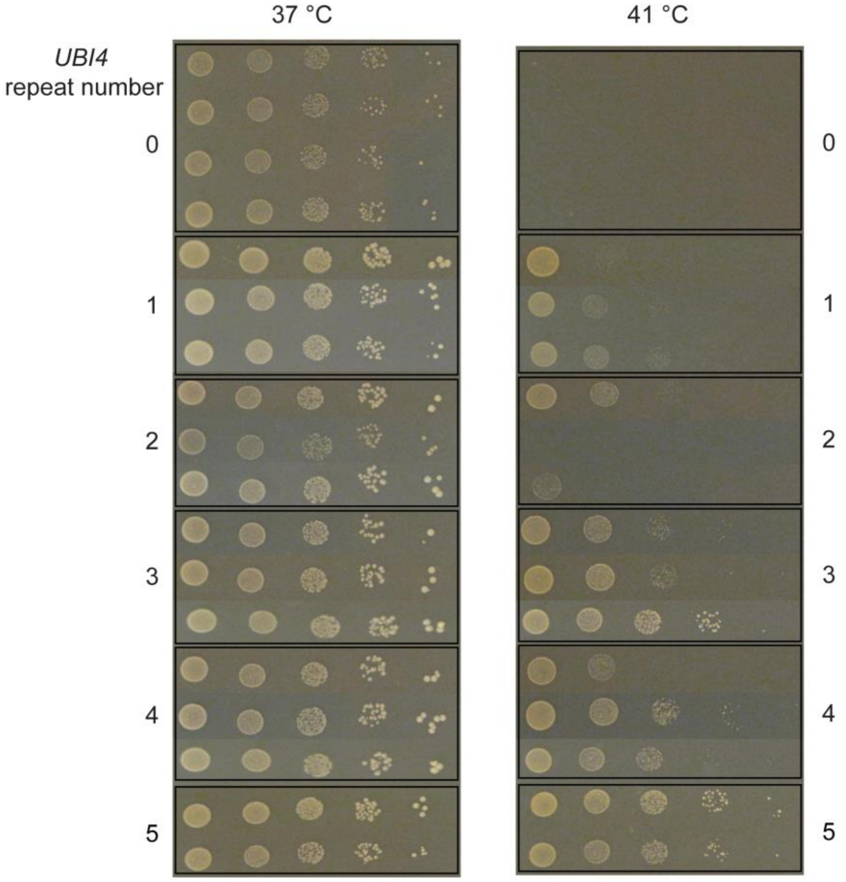

Supplementary Figure 2. Fitness of the *UBI4* repeat variants at elevated temperatures depends on the number of ubiquitin repeats

Multiple biological replicates of each *UBI4* repeat variant were 10-fold serially diluted and spotted on nitrogen-starved (SLAD) medium. The plates were incubated at 37°C or 41°C. The replicates were positioned in a random order on multiple plates to account for position effects on growth.

Supplementary Figure 3

A

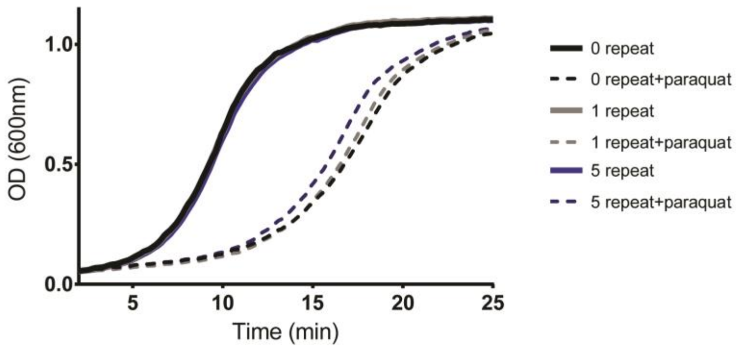

B

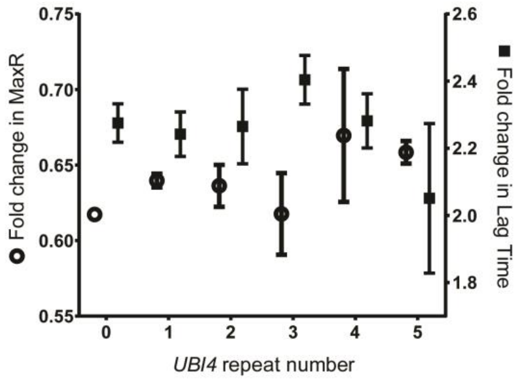

Supplementary Figure 3. Fitness of *UBI4* repeat variants in the presence of oxidative stress depends on the number of ubiquitin repeats.

The *S. cerevisiae* S288c *UBI4* variant encoding 5 ubiquitin units displays better growth in the presence of 2.5 mM paraquat (oxidative stress) than *UBI4* variants encoding 0 or 1 ubiquitin unit.

A. Representative growth curves of null, 1-repeat, and 5-repeat variants with and without paraquat.

B. Fold changes (oxidative stress/YPD) in maximal growth rate (MaxR) and lag time for all repeat variants. Data points represent mean ± SD of 3 biological replicates, each with 2 technical replicates.

Supplementary Figure 4

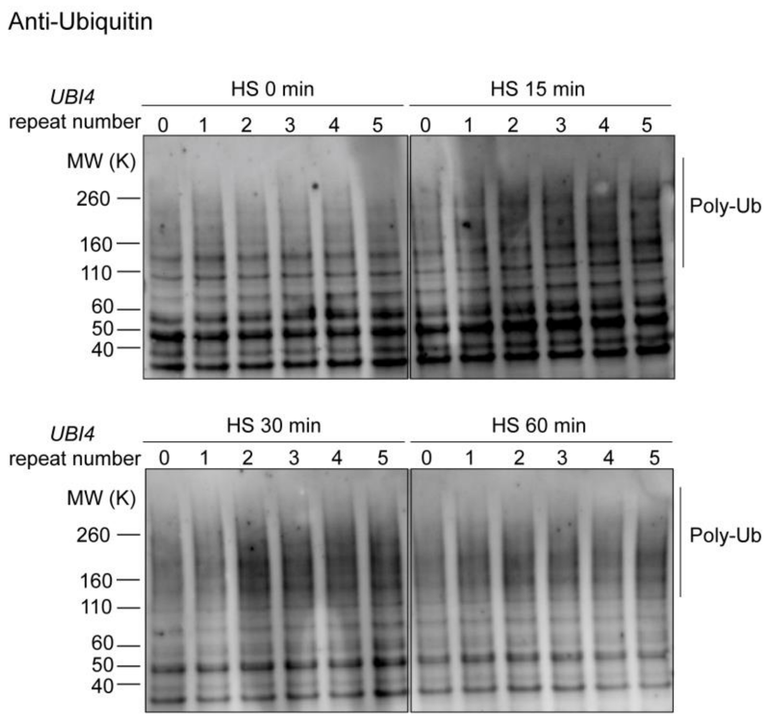

Supplementary Figure 4. Differences in heat shock-induced polyubiquitination of proteins between the *UBI4* repeat variants

Ubiquitination levels in the soluble fractions of the *UBI4* repeat variants during a heat shock (HS) time series were analysed by western blot using anti-ubiquitin antibody. Uncropped images of the blots are shown in Supplementary Fig. 5.

Supplementary Figure 5

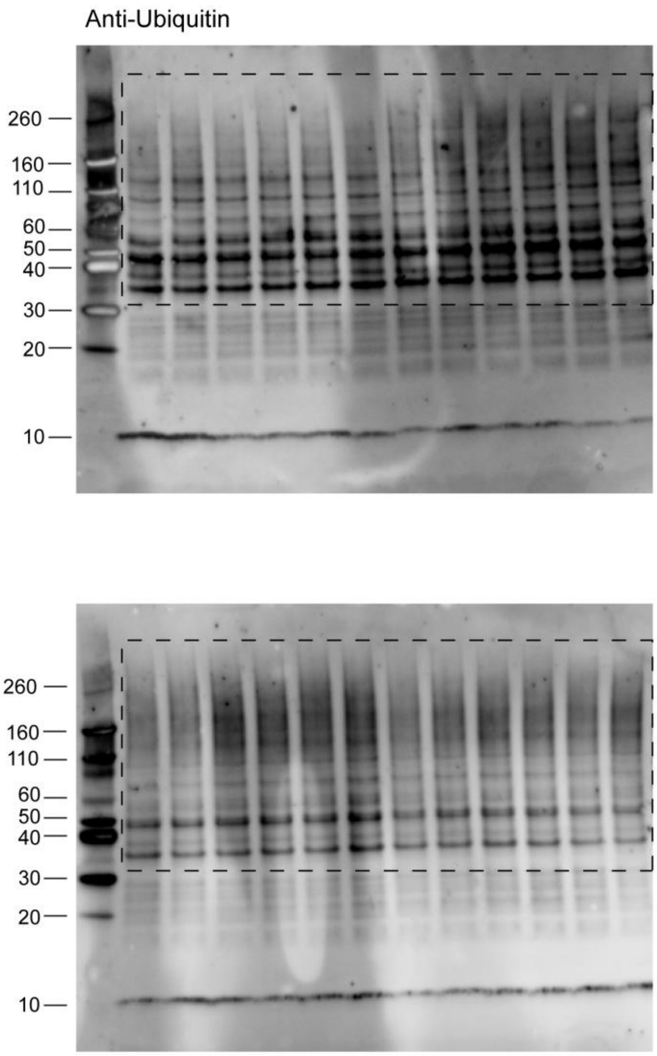

Supplementary Figure 5. Uncropped images of the western blots shown in Supplementary Fig. 4.

The cropped areas shown in Supplementary Fig. 4 are marked.

Supplementary Figure 6

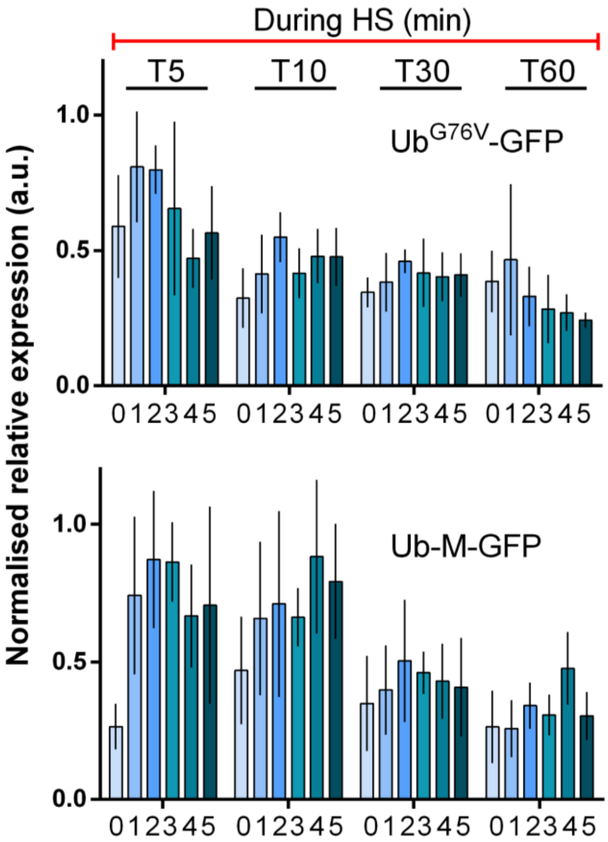

Supplementary Figure 6. Differences in Ub-GFP half-life are not due to differences in substrate expression

Time series of *GFP* expression in the repeat variants (carrying a *UBI4* gene encoding either 0, 1, 2, 3, 4 or 5 ubiquitin units) expressing Ub^{G76V}-GFP (a substrate for UPS-mediated proteolysis) or Ub-M-GFP (stable GFP) during a sustained heat shock (HS) at 44°C. *GFP* transcripts were measured by real-time quantitative PCR (RT-qPCR) using primers that specifically anneal to GFP. For each sample, GFP expression was normalized to the expression of the *ACT1* gene and to timepoint zero. Statistical analysis of the data can be found in

Supplemental Dataset 3. Data are mean of 4 biological replicates. Data points represent mean ± SD.

Supplementary Figure 7

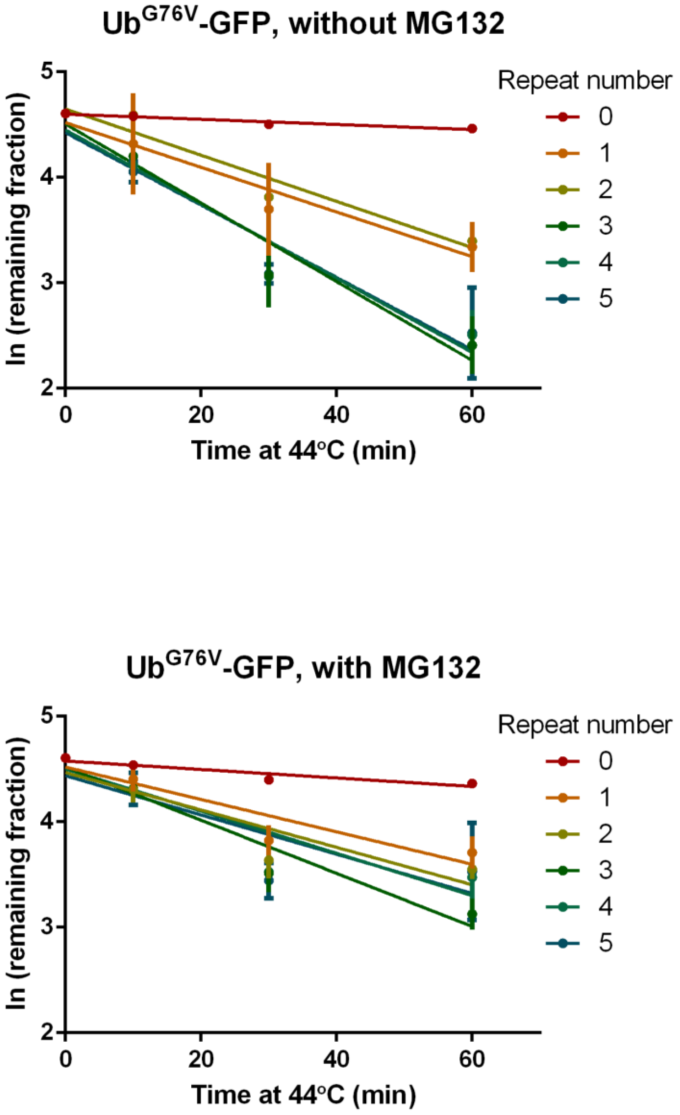

UBI4 repeat number	fold increase in GFP 1/2 life
0	0.61
1	1.40
2	1.25
3	1.48
4	1.83
5	2.06

Supplementary Figure 7. Quantitative protein turnover measurements during heat shock in the presence of a proteasome inhibitor confirms that Ub^{G76V}-GFP degradation is proteasomal.

UBI4 repeat variants expressing Ub^{G76V}-GFP (a substrate for UPS-mediated proteolysis) were used to measure the UPS activity during heat shock (44°C). Cells were grown at 30°C until exponential phase in SC medium with 2% galactose to induce the expression of the fluorescent reporters. Cultures were then split, and 100 µM MG-132 (final concentration) was added to

inhibit proteasomal activity. After 5 min incubation at 30°C, all cultures were shifted to 44°C and samples were taken immediately before the temperature shift (T0) and at multiple time points afterwards for flow cytometry analysis. For each time point, the fraction of fluorescent cells over the total cell population was calculated. We then fit the fraction to a first-order decay function to calculate the degradation rate constant K_{deg}. Protein half-lives were calculated using the formula $T_{(1/2)} = \ln(2)/K_{deg}$. Table depicts the fold increase in protein half-lives (MG-132 compared to no MG-132).

Supplementary Figure 8

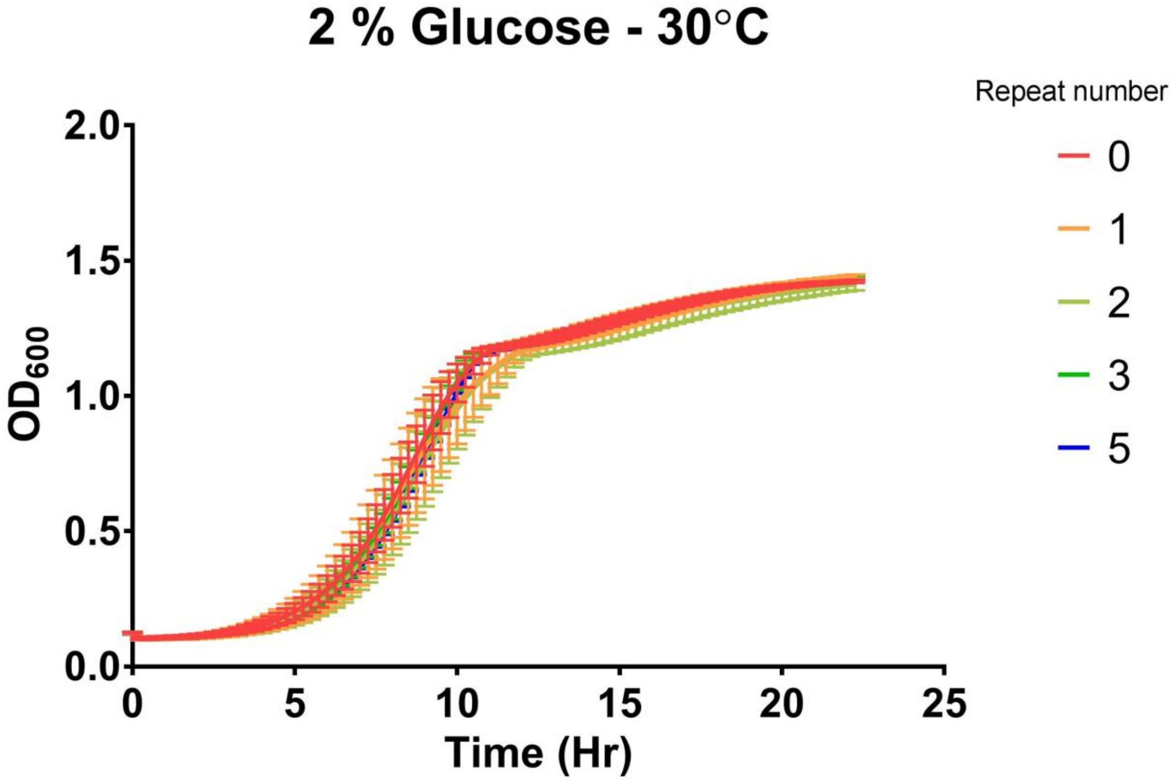

Supplementary Figure 8. ***UBI4*** **repeat variants display similar growth profiles on glucose.**

UBI4 repeat variants show similar growth profiles when grown on 2% glucose as a carbon source. Data are mean of 3 biological replicates. Data points represent mean ± SEM.

Supplementary Methods

Strain construction

The *S. cerevisiae* UBI4 variants were engineered in the S288c background. To create the *UBI4* tandem repeat number variants we first amplified the hygromycin B resistance gene, *Hph*, using primers 3979 - 3980 and plasmid pAG34 (Addgene). The resulting PCR product contains 60 bp overhangs that allow homologous recombination 150 bp downstream of the *UBI4* open reading frame. This strain (RG402) will be the WT *UBI4* strain containing five tandem ubiquitin repeats. Next, we amplified different PCR products using genomic DNA from RG402, the reverse primer 3980 and a series of forward primers (3981, 3982, 3983, 3984) each designed to amplify a *UBI4* open reading frame with different number of tandem ubiquitin moieties, and the downstream *Hph* marker. These PCR products were used to transform a fresh S288c strain and the transformants were selected for hygromycin B resistance. The forward primers were designed in a manner that allowed us to ascertain that the last ubiquitin unit in every *UBI4* repeat variant was the same and contains the Asn residue at the carboxy terminus. This is of vital importance in order to prevent unprocessed polyubiquitin from being conjugated to proteins. All engineered *UBI4* repeat variants were verified by sequencing using primers (3996 - 3997) flanking the *UBI4* open reading frame.

To construct the mono-ubiquitin copy number variants we first amplified a loxP-flanked *KanMX* marker (conferring resistance to G418) from plasmid pUG6 (Euroscarf) using primers 4375 – 4376 and inserted this cassette downstream of the *UBI4* gene in the S288c background to obtain strain RG614. Using primers 3982 – 4376 and gDNA from RG614, we amplified a PCR product that includes one *UBI4* ubiquitin moiety and the downstream *KanMX* cassette, which we then transformed into a new S288c strain to yield strain RG621. Upon expression of the Cre recombinase [1], the *KanMX* module was efficiently excised from strain RG621 yielding

the G418 sensitive strain RG623. Using gDNA from strain RG621 and primers 4376 – 4377 containing adequate 60bp overhangs, we amplified the single mono-ubiquitin moiety plus its promoter (400bp upstream of ATG) and the downstream *KanMX* cassette and transformed the PCR product into strain RG623. The resulting G418 resistant strain, RG625, thus contained 2 tandem copies of the *UBI4* promoter and its mono-ubiquitin moiety and a single downstream *KanMX* cassette. We again expressed the Cre recombinase in RG625 to excise the *KanMX* marker and yield strains RG710 and RG713. We next amplified the *UBI4* promoter plus the single ubiquitin moiety and the downstream hygromycin B resistance cassette from strain RG525 using primers 4535 – 4536. The resulting PCR product contains 60 bp overhangs that allowed the insertion of the third copy of the mono-ubiquitin moiety plus its *UBI4* promoter in a neutral location on chromosome XIII in strain RG713, creating strains RG727 – RG728. The 3rd copy of the mono-Ub was inserted in another location of the genome because of the high chance of homologous recombination happening in the *UBI* sequence itself (which is much longer than the flanking sequences) and this would have resulted in replacing one of the existing two copies. We verified the various insertions by sequencing. Additionally, we verified the mono-ubiquitin copy numbers by quantitative real-time PCR (RT-qPCR) on genomic DNA from all the engineered copy number variants.

To construct the strain containing only one ubiquitin unit containing a K48R point mutation (refered to as 1 (K48R) in text), we first set up a PCR using gDNA of RG621 as template and primers PYY117-PYY118. A second PCR was set up using gDNA of RG621 as template and primers PYY119-PYY120. Next, a fusion PCR was performed, using these two PCR products as template and primers PYY117-PYY120, to obtain a mono-repeat ubiquitin moiety containing a K48R mutation linked to a *KanMX* resistance cassette. This PCR product was subsequently transformed to RG623, to create strain YY399. Presence of the K48R mutation was verified by sequencing, using primers PYY127 and PYY128.

All primers and strains are listed in **Supplementary Dataset 2** and **3**.

Various natural *Saccharomyces* species and strains were obtained from [2, 3] and can be found in **Supplementary Dataset 2**.

Supplementary References

1. Guldener, U., et al., *A new efficient gene disruption cassette for repeated use in budding yeast.* Nucleic Acids Res. **24**(13): p. 2519-24 (1996).
2. Gallone, B., et al., *Domestication and Divergence of Saccharomyces cerevisiae Beer Yeasts.* Cell. **166**(6): p. 1397-1410 e16 (2016).
3. Steensels, J., et al., *Large-scale selection and breeding to generate industrial yeasts with superior aroma production.* Appl Environ Microbiol. **80**(22): p. 6965-75 (2014).
